# A Review on the Protective Effects of Probiotics against Alzheimer’s Disease

**DOI:** 10.3390/biology13010008

**Published:** 2023-12-22

**Authors:** Vibhuti Mishra, Dhananjay Yadav, Kavita Singh Solanki, Bhupendra Koul, Minseok Song

**Affiliations:** 1 School of Studies in Biochemistry, Jiwaji University, Gwalior 474003, India; vibhu.mishra2007@gmail.com; 2 Department of Life Science, Yeungnam University, Gyeongsan 38541, Republic of Korea; dhanyadav16481@gmail.com; 3 Department of Neuroscience and Pharmacology, Iowa Neuroscience Institute, University of Iowa, Iowa City, IA 52242, USA; kavita-solanki@uiowa.edu; 4 School of Bioengineering and Biosciences, Lovely Professional University, Phagwara 144411, India; bhupendra.18673@lpu.co.in

**Keywords:** Alzheimer’s disease, gut–brain axis, gut microbiome, probiotics, neurodegeneration, dysbiosis, neurofibrillary tangles, neurodegenerative disorders

## Abstract

**Simple Summary:**

Alzheimer’s disease (AD) is one of the most common neurodegenerative disorders in older adults. It is characterized by difficulty in writing and speech, weak memory, and struggle with cognition. Bidirectional communication between the gut and brain via the gut–brain axis plays a very important role in normal brain functioning. Dysbiosis has been linked to various neurodegenerative disorders; hence, a healthy gut microbiota is essential for normal brain function. Probiotics can prove to be effective therapeutic agents against AD as they restore gut–brain homeostasis.

**Abstract:**

This review summarizes the protective effects of probiotics against Alzheimer’s disease (AD), one of the most common neurodegenerative disorders affecting older adults. This disease is characterized by the deposition of tau and amyloid β peptide (Aβ) in different parts of the brain. Symptoms observed in patients with AD include struggles with writing, speech, memory, and knowledge. The gut microbiota reportedly plays an important role in brain functioning due to its bidirectional communication with the gut via the gut–brain axis. The emotional and cognitive centers in the brain are linked to the functions of the peripheral intestinal system via this gut–brain axis. Dysbiosis has been linked to neurodegenerative disorders, indicating the significance of gut homeostasis for proper brain function. Probiotics play an important role in protecting against the symptoms of AD as they restore gut–brain homeostasis to a great extent. This review summarizes the characteristics, status of gut–brain axis, and significance of gut microbiota in AD. Review and research articles related to the role of probiotics in the treatment of AD were searched in the PubMed database. Recent studies conducted using animal models were given preference. Recent clinical trials were searched for separately. Several studies conducted on animal and human models clearly explain the benefits of probiotics in improving cognition and memory in experimental subjects. Based on these studies, novel therapeutic approaches can be designed for the treatment of patients with AD.

## 1. Introduction

The human body harbors a complex and dynamic population of microorganisms, which is collectively known as the microbiota [1]. The digestive system alone contains approximately 100 trillion bacteria [1]. Microbiota aids in the fermentation of food fibers, prevents pathogenic infections, and is crucial for maintaining human health [2].

Microbiomes have recently been extensively explored using various technologies. Studies on the human microbiome have been greatly aided by initiatives such as the Human Microbiome Project (HMP) and MetaHIT (Metagenomics of the Human Intestinal Tract) [3,4].

A significant amount of information regarding microbiome composition in various environments has been made available using high-throughput sequencing technology [5,6]. Dysbiosis, an imbalance in the gut flora, has been reported to be one of the major causes of several human disorders [7]. Interestingly, dysbiosis also plays a role in a number of age-related disorders, demonstrating that the impact of the microbiome is not restricted to the oro-gastrointestinal tract [7]. Disturbances in the gut microbiome have been linked to various neurodegenerative diseases such as Alzheimer’s disease (AD), Parkinson’s disease (PD), amyotrophic lateral sclerosis (ALS), and multiple sclerosis (MS) [8,9,10,11].

According to Castelli et al. and Cenini et al., neurodegenerative disorders are a diverse group of severe impairments characterized by changes in genes, elevated levels of reactive oxygen species (ROS), mitochondrial damage, altered calcium (Ca^2+^) homeostasis, protein buildup, ongoing inflammation, and/or neuronal damage in specific regions of the brain [12,13]. Numerous illnesses connected to neurological diseases have been linked to increased oxidative stress and moderate chronic inflammation. Ca^2+^ ions are crucial for various biological processes [14,15], and their cellular levels are regulated by various mechanisms. A disruption of the Ca^2+^ equilibrium causes a buildup of ions inside the cells and organelles, which culminates in the development of neurodegenerative disorders.

Activation of numerous harmful pathways and degradation of cellular energy synthesis are caused by mitochondrial dysfunction [16]. Neurodegenerative diseases cause clinical problems such as cognitive decline and other clinical ailments [13]. Patients with AD and PD, vascular and mixed dementia, and dementia linked to Lewy bodies demonstrate a loss in cognition [13,17]. ALS, Huntington’s disease (HD), and PD are associated with motor dysfunction. Aging is a risk factor for all of these illnesses [13,17]. The severity of these illnesses increases with age, leading to significant social and economic repercussions [18,19].

Neurodegenerative illnesses occur and progress due to genetic and lifestyle factors [20,21] and are significantly influenced by gene–environment interactions [22,23]. The human gut is a reservoir of several bacterial species, and the human body is home to a variety of microorganisms, including bacteria, viruses, archaea, and microeukaryotes [24]. Interestingly, patients with neurological, autoimmune, metabolic, and cancer illnesses have distinct gut microbiota compared with healthy individuals [25,26,27]. The enteric nervous system (ENS) and central nervous system (CNS) communicate bidirectionally through the gut–brain axis [28,29].

Vagal and spinal afferent fibers connect the brain and gastrointestinal tract, and parasympathetic and sympathetic efferent nerve fibers facilitate communication between the two [29,30]. According to several studies [30,31,32], the altered microbiota of an unhealthy gut interferes with the communication between the brain and gut, which can result in psychiatric diseases such as neurodegenerative disorders, autism, and anxiety. According to Breit et al. and Srikantha and Mohajeri (2019), metabolic diseases are underpinned by malfunction of the gut–brain axis [30,31].

The composition of the gut microbiome controls immunological responses, resulting in the release of chemokines and cytokines. The gut–brain axis and intestinal cells communicate with each other through metabolic and neuroendocrine pathways [33,34]. The gut microbiome affects ENS function [34]. Neurotransmitters including catecholamines, fatty acid derivatives, and amino acids are released in the lumen of the intestine [33,35]. Bacteria activates the sympathetic nervous system by releasing propionic acid and acetic acid, which in turn affects the learning ability and memory of the host. Probiotics, according to FAO, are live microorganisms that provide positive benefits when taken orally by the host [36]. Neurodegenerative illnesses have been shown to benefit from probiotic use in terms of both therapy and prevention [37,38,39]. Studies have shown that probiotic consumption delays the onset of neurodegenerative diseases such as MS and PD. Probiotics improve the motor and non-motor impairments of these diseases by altering biochemical processes such as inflammatory and non-inflammatory pathways, along with oxidative stress. They also alter the intestinal microbial composition [40,41]. Probiotics may boost the gut flora, which controls the inflammatory response and acts as a defense mechanism against external pathogenic bacteria [1]. They are particularly effective in reducing oxidative stress and inflammation, which are the two main causes of neurodegeneration [1]. PD and AD are two neurodegenerative conditions associated with a decline in brain-derived neurotrophic factor (BDNF) [38]. BDNF, a protein that aids in the survival and proliferation of neurons, is produced by several probiotics [42]. This review highlights the importance and mechanisms of action of probiotics against neurodegenerative illnesses, particularly AD. It also focuses on the challenges and future prospects of probiotics in AD treatment regimes.

## 2. Gut–Brain Axis and Its Significance in Neurodegenerative Disorders

The gut–brain axis is a two-way communication system that connects the brain to the digestive system. This intricate biochemical pathway aids in interaction between the gastrointestinal (GI) tract and CNS via neurological, humoral, endocrine, and immunological connections [43].

Various bioactive metabolites are produced by gut bacteria during metabolic activities [42]. Enterohepatic circulation of these metabolites allows them to eventually enter the bloodstream [44,45]. Nuclear magnetic resonance (NMR) and mass spectrometry-based metabolomics can aid in the characterization of bodily fluids and metabolites, which can provide clues regarding their correlation with the disease phenotype [44,45]. Metabolomics is a technology that thoroughly analyzes the metabolites in a biological specimen and aids in diagnosing various diseases [46]. Mechanistic connections between gut microbiota and the brain can be established by examining the microbiome, metabolome, and host phenotypes [47,48].

Numerous neuromodulators and neurotransmitters, including acetylcholine, dopamine, serotonin, and short-chain fatty acids, are metabolized by the gut bacteria [49,50]. It has been suggested that probiotic microbes such as *Lactobacillus*, *Bifidobacterium*, *Bacillus*, and *Saccharomyces* produce neurotransmitters. Initial research has demonstrated that bacterial engagement in these processes can also alter the amount of neurotransmitters involved in synaptic plasticity, including brain-derived neurotrophic factor, serotonin, and N-methyl-d-aspartate receptor function [51].

When the composition of the gut microbiota or the chemicals released by the gut are disturbed, the gut–brain axis is modulated, which reportedly leads to regulation of social behavior, mood, memory, and cognition [43,52,53]. Dysbiosis also creates toxic misfolded proteins that promote synaptic loss, neurodegeneration, and cellular malfunction [54,55].

The hypothalamic–pituitary–adrenal axis (HPA axis), which releases pro-inflammatory cytokines and affects several physiological systems, including the immune system and inflammatory pathways linked to the endocrine glands, plasma, and brain, is one of the most crucial parts of the gut–brain axis [56,57]. The HPA pathway (involving the hypothalamus, pituitary, and adrenals) of the gut–brain axis is an endocrine regulatory route that regulates the CNS to govern metabolic and immunological homeostasis. Under stressful conditions, the HPA axis is activated, which increases the circulation of corticosteroids, thereby inhibiting various immune activities [58]. Herman et al. reported that the HPA axis releases glucocorticoids that affect immune cells and mediators [59]. According to Chovatiya and Medzhitov (2014), the constant influence of the HPA axis on peripheral inflammation is linked to its baseline activity [60]. The release of glucocorticoids is triggered by a variety of immunological, mental, and physical stressors [61,62]. Inflammatory Bowel Disease (IBD) is a long-term inflammatory condition of the digestive system, wherein the dysbiotic microbes cause intestinal tissue injury, thereby perpetuating immune responses and eventually affecting the HPA axis [63]. The involvement of gut microbiota and inflammation in IBD has recently attracted considerable attention [64]. To understand the pathophysiology of IBD, it is crucial to investigate both the immunoinflammatory response and peripheral mediators of inflammation [65]. Glucocorticoids, mineralocorticoids, and catecholamines are associated with the HPA pathway. As evidenced by a decrease in the *Bacteroides* genus and an increase in the *Clostridium* genus, increased corticosterone levels in stressed mice lead to dysbiosis [66,67,68]. The pro- and anti-inflammatory properties of glucocorticoids affect the peripheral and central nervous systems. Damage to the HPA axis causes conditions like multiple sclerosis, rheumatoid arthritis, and IBD [69]. The gut microbiome produces several endocrine signaling chemicals, both directly and indirectly. SCFAs, which are produced by the gut microbiota, contain signaling molecules that encourage the production of peripheral neurotransmitters and prevent the generation of ROS [70]. SCFAs exert their effects by suppressing apoptosis and increasing FoxP+ transcription. The HPA axis is activated by the gut microbiota, which causes cortisol production. Cortisol suppresses the inflammatory response and affects neural pathways [71]. Numerous stressors have reportedly had an impact on the *Clostridium*, *Bacteroides*, and *Lactobacilli* populations in animal models. Stress also affects the digestive system integrity. This is most likely because stress-related catecholamines promote the growth of Gram-negative bacteria [43,72]. Stress reportedly activates the HPA axis in germ-free mice [73]. In addition to blood indicators, stress affects germ-free mice, as has been demonstrated by the activation of genes linked to the HPA axis response [74]. Researchers found that stress changes the gut microbiome of mice and rats raised in a germ-free environment. The gut microbiome also affects stress response and brain neurochemistry. When exposed to acute stress, germ-free mice display an augmented HPA axis response, producing more plasma adrenocorticotropic hormone (ACTH) and corticosterone. When commensal bacteria were administered to the mice, their levels of ACTH and corticosterone were restored [75]. These results indicate that the activity of the HPA axis, including the plasma levels of glucocorticoids, is significantly influenced by the gut flora. The brain and/or other peripheral organs receive signals produced by the bacteria and produce glucocorticoids. Glucocorticoids can also be secreted from tissues outside the adrenal cortex. This can occur in the intestine either through extra-adrenal glucocorticoid synthesis or via regeneration of biologically active corticosterone, glucocorticoids, or cortisol from inactive 11-oxo derivatives by 11-hydroxysteroid dehydrogenase type 1 (11HSD1) [57,76]. Thus, the gut bacteria affects glucocorticoid steroidogenesis. The HPA axis, brain activity, steroidogenesis in the adrenal glands, and regeneration of glucocorticoids are all affected by cytokines, innate immune receptors, and chemokines expressed by enterocytes via 11 HSD1 [77].

SCFAs are produced by gut microbes such as *Eubacterium rectala*, *Clostridium leptum*, and *Faecalibacterium prausnitzil* by suppressing pro-inflammatory cytokines that play a crucial role in triggering neurodegeneration [78]. Short-chain fatty acids from microorganisms are formed during the fermentation of bacterial cells and have significant neurological effects [70]. They function as serotonin and other neuropeptide modulators, which strengthens the multistage gut–brain axis communication. According to Nagahara and Tuszynski (2011), the production of excessive amounts of SCFAs has a significant impact on the behavioral responses and brain health of humans [79]. Tryptophan, an important amino acid, plays a key role in the synthesis of numerous neurotransmitters, including serotonin. An imbalance in tryptophan levels reportedly leads to gastrointestinal and brain abnormalities that can result in mood disorders, neurodegeneration, and cognitive decline [80,81,82]. GABA is a by-product of bacterial metabolism and a significant inducer of neuronal stimulation [83]. Various pathological imbalances caused by improper GABA regulation play a significant role in neurotoxicity, which in turn results in chronic neurological illnesses [84]. The action of GABA provides evidence that the gut bacteria regulates brain chemistry.

## 3. AD

AD is one of the most prevalent types of dementia worldwide. It can be classified into two categories: sporadic and familial. It can also be categorized into two types based on age: early- and late-onset. Neuronal dysfunction in particular brain regions is the primary factor that contributes to AD progression [85,86]. The main signs of AD are discomfort when writing or speaking, decline in memory, and challenges in problem solving. The symptoms tend to worsen with disease progression [87,88].

Two proteins, ‘Tau’ and ‘amyloid beta’, have been found to accumulate in several brain tissues of people with AD [89]. Research on AD has shed light on the fact that the exterior of the nerve cell is where amyloid builds up. When the Tau protein transforms into helical pieces and these fragments connect with one another, neurofibrillary tangles are created [90]. Amyloids diffuse into the synaptic cleft following oligomerization, obstruct neuronal signal transmission, and ultimately render the cell nonfunctional [91]. Microtubule tubulin polymerization is mediated by tau proteins. Under normal conditions, tau proteins possess 2–3 phosphates; however, under abnormal conditions, 5–9 phosphate groups per molecule of tau are formed after polymerization.

The loss of tau–tubulin protein affinity caused by hyperphosphorylation affects the development and integrity of microtubules [92]. The gut microbiota–brain axis, which is bidirectional and plays a critical role in brain development, has been linked to the brain in numerous studies. This association is known as the gut microbiota–brain axis [93]. Lower levels of BDNF have been reported in patients with AD. The gut microbiota can affect the regulation of N-methyl-D-aspartate receptor (NMDAR), BDNF, and neuroinflammation, along with the neuroendocrine, direct neuronal, and immunological pathways [94]. Lower levels of BDNF expression have been reported in the cortex and hippocampus of germ-free animals [95,96].

## 4. Gut Microbiota in AD

**Figure 1:**
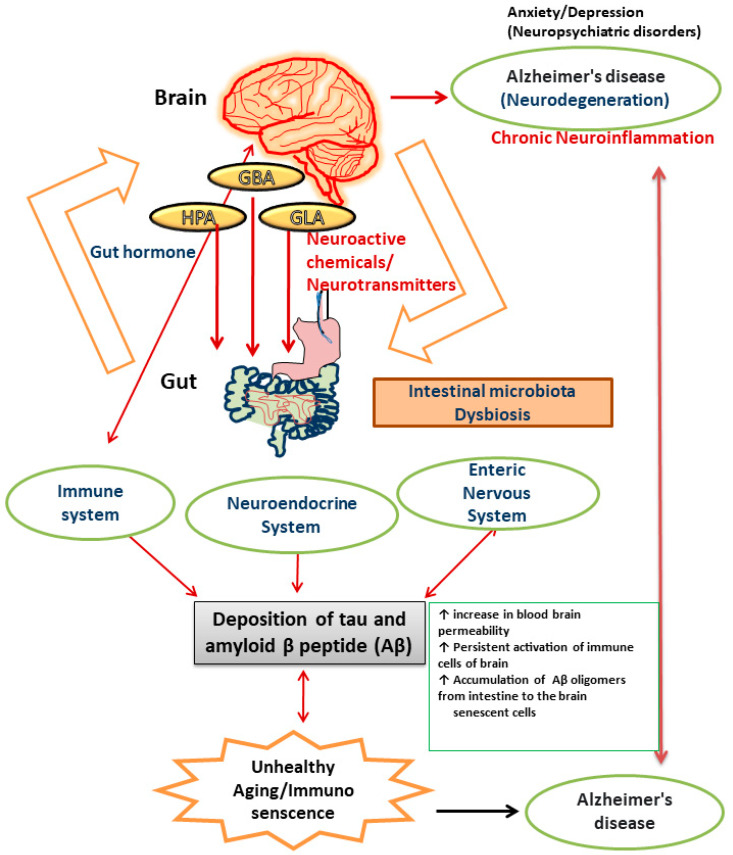
Pictorial representation of the components of gut–brain axis affected by dysregulated gut microbiota that results in AD.

There is numerous evidence that the gut microbiota plays a role in the neuropathology of AD and has an impact on a number of processes linked to its etiology, including neuroinflammation, neurotransmitter dysregulation, Aβ abnormality, oxidative stress, and tau phosphorylation [97]. When the microbiota composition is disturbed, neuronal pathways become dysregulated and are linked to an increase in blood–brain barrier permeability, which causes neuronal cell death, neuroinflammation, and ultimately AD [98]. The brain is comprised of cells like neurons, microglia, astrocytes, endothelial cells, and oligodendrocytes. Accumulation of inflammatory chemicals resulting from the activation of innate and acquired immunity causes inflammation in the brain. The gut microbiota and expression of central immune cells are reportedly closely related [99]. A study reported that administration of antibiotics to germ-free mice resulted in an impairment in immune maturation in terms of microglia maturation and altered sensitivity to bacterial stimuli [100]. Alterations in the gut microbiota can make the intestine more permeable and activate proinflammatory cytokines, which in turn induces movement of Aβ oligomers from the intestine to the brain. The observation that neuroinflammation and AD are induced by amyloid was made after injecting Aβ1–42 oligomers into the stomach wall of mice [101]. Neuroinflammation and brain dysfunction can be induced by proinflammatory cytokines produced as a result of systemic inflammation. Recent studies on germ-free (GF) animals has shown that microbial colonization in the intestinal lining is the crucial event for the development, proliferation, and maturation of cellular inputs in both ENS and CNS [94]. The immunological aberrations associated with aging are now more commonly known as immuno-senescence of the brain [101]. Alterations in the diversity of gut microbiota constituents and metabolic components, including gut hormones, lead to impairments in different organs (brain, liver, lungs, heart, joints, and adipose tissues) in the human body [102]. Moreover, several factors have been reported to be responsible for chronic inflammation during ageing. These include upregulated pro-inflammatory cytokine secretion, continuous activation of immune cells of the brain like glia and astrocytes (reactive microgliosis and astrogliosis), alterations in the intestinal gut microbiome, and increased permeability of the intestinal membrane [103] [Figure 1].

When the intestinal barrier function is disrupted, the interaction between lipopolysaccharide (LPS) of gut bacteria and the Toll-like receptor 4 signaling pathway stimulates immune system cells [104,105]. The pathogenesis of AD is contemplated to result from polymerization of soluble forms of Aβ into insoluble forms of protein. Studies have reported a connection between the composition of gut microbiota and accumulation of Aβ in the brain [106]. The gut microbiota of APPswe (transgenic) mice has been shown to alter, and this alteration correlates with an increase in the expression of the amyloid precursor protein and stimulation of the MAPK signaling pathway, which results in amyloid deposition [107] and activation of astrocytes, which plays a significant role in the pathogenesis of AD [108]. The innate immune system in the CNS depends heavily on the microglia. Due to the absence of helpful host microbiota, it was found that deficiencies in microglial characteristics emerged in germ-free animals [100]. Microglia creates a barrier of defense surrounding amyloid deposits, preventing fresh Aβ from adhering to already-formed plaques [109]. The microglia cannot remove the amyloid build up in conditions of persistent inflammation. The composition of the gut microbiota is altered in APPswe mice, which leads to a decrease in Aβ deposition [110]. Pro-inflammatory cytokines like IL-22 and IL-17A are released as a result of pathogens secreting Aβ. These cytokines can cross the blood–brain barrier (BBB), activate the immune system, and ultimately cause chronic neurodegenerative diseases like AD [111,112]. The protein tau, which is linked to microtubules, creates neurofibrillary tangles of paired helical segments in AD as a result of aberrant phosphorylation. The phosphorylation is altered by the gut microbiota, which contributes to the pathogenesis of AD. Trimethylamine N-oxide (TMNO) has been detected in higher concentrations in the cerebrospinal fluid (CSF) of patients with mild cognitive impairment and AD. CSF TMNO promotes tau protein hyperphosphorylation, which plays a significant role in the pathology of AD [113,114]. Fecal microbiota transplantation from WT mice into the transgenic mouse model (ADLP^APT^) has been shown to reduce tau pathology and memory impairment [115]. This transgenic mouse model (ADLP^APT^) has a pathology similar to that of AD with amyloid plaques and neurofibrillary tangles.

In neurodegenerative disorders, neurotransmitters such as serotonin (5-HT), acetylcholine (Ach), noradrenaline, dopamine, histamine, serotonin, and GABA have the power to alter the immune system pathways that affect memory, behavior, and learning. Studies have reported that gut bacteria have the ability to create neurotransmitters and significantly impact the modulation of the gut–brain axis [51,116]. The brains of patients with AD have significantly lower levels of GABA and glutamate neurotransmitters, which is indicative of impaired synaptic function and neuronal transmission [117]. GABA is an inhibitory CNS neurotransmitter that is known to be produced by bacteria like *Streptococcus*, *Lactobacillus*, and *Bifidobacterium* [51]. A recent study reported that consuming a large amount of dietary fiber increases the expression of 5-HT, a neurotransmitter with critical roles in the control of sleep, mood, appetite, and sexual function. In addition, 5-HT also inhibits neuroinflammation. *Lactobacillus*, *Streptococcus*, and *Escherichia coli* generate 5-HT in the gut [83]. *Serratia marcescens*, *Proteus vulgaris*, *Escherichia coli*, and *Bacillus* species produce catecholamines, including noradrenaline and its precursors [51,118].

According to Chen et al., *Staphylococcus* can convert the precursor L-3,4,-dihydroxy-phenylalanine (L-DOPA) into dopamine in the human colon [119]. Dopamine, norepinephrine, and catecholamine levels in patients with AD have been reported to alter [120]. Increased oxidation has been observed in the brains of patients during the progression of AD. Manoharan et al. reported that the gut microbiota may modify the amount of ROS or interfere with the antioxidant system, which could affect the oxidative state in patients with AD [121]. Oxidative stress can lead to acceleration of Aβ deposition and the start of an oxidative response [122]. A study reported that the twofold transgenic mice model of AD demonstrated increased oxidative stress and Aβ deposition [123]. An increase in tau hyperphosphorylation increases Aβ and causes more neuronal death. Oxidative stress is thought to be a pathogenic hallmark in the development of AD [124,125].

Probiotics significantly affect the progression of AD. Bacteria from the genera *Proteobacteria*, *Actinobacteria*, *Firmicutes*, and *Verrucomicrobia* are significantly reduced in patients with AD. *Bacteroidetes* and *Tenericutes* are also diminished [126]. The increased deposition of Aβ in the cerebrum occurs due to this imbalance in microbial constitution [127]. Intrinsic pathogen colonization may result from changes in the gut microbiota. This causes the gut permeability to increase, which disrupts the gut–brain axis system. An increase in immune hemoglobin migration to the brain further modifies the development of AD. According to Wu et al., this results in the start of TNF-JNK-mediated neurodegeneration in AD [128].

The gut microbiota changes in patients with AD demonstrating brain amyloidosis and cognitive impairment. This is accompanied by an increase in pro-inflammatory bacteria such as *Escherichia* spp. or *Shigella* spp. and a decrease in anti-inflammatory bacteria such as *Enterococcus rectale* [129]. Therefore, it can be inferred that changes in bacterial strains significantly affect AD development [130].

## 5. Probiotics: Mechanism of Action in AD

Probiotics exert their positive benefits through a variety of methodologies, including creation of SCFAs, release of bacteriocin, immunomodulation, and their impact on the gut–brain axis [131]. SCFAs are saturated fatty acids produced in the stomach as a result of dietary fibers. Verbeke et al. reported that fermentation mediated by *Bacteriodes*, *Clostridium*, *Lactobacillus*, *Bifidobacterium*, and *Eubacterium* species produces metabolites such as acetate, butyrate, and propionate [132]. SCFAs affect brain function via three main pathways, neuronal factors, endocrine route, and immunological modulation. When the immune activity is modulated, SCFAs increase barrier integrity and maintain mucus production, which affects barrier performance and intestinal mucosal immunity. Additionally, when immunomodulation occurs, cytokines are released, which affects immune cell differentiation and proliferation [133]. Pro-inflammatory cytokines (such IL-1, IL-6, and TNF-α) are suppressed as a result of this interaction, which also results in production of an anti-inflammatory response. Additionally, SCFAs can enhance the expression of tight junction proteins which influences the integrity of BBB. This occurs when SCFAs use monocarboxylate transporters to penetrate the BBB [134]. SCFAs prevent the death of neuronal cells in the CNS by affecting microglial cell shape. SCFAs act as endocrine signaling molecules by promoting the release of gut hormones. Stimulation by acetate and propionate contributes considerably to the release of glucagon-like peptide 1 (GLP-1) and peptide YY (PYY) via G-protein-coupled receptors in murine colonic cells [135].

GLP-1 functions as a neuroprotective agent by preventing cell death and neuronal apoptosis [136,137]. In Alzheimer’s dementia, a different molecule known as neuropeptide Y exerts neuroprotective effects by reducing oxidative stress, inhibiting caspase-3 and caspase-4 activities, and activating the PI3K-XBP1-induced Gip78/BiP pathway, among other mechanisms [138]. SCFAs affect the neurotransmitter levels in the body. Studies have shown that the synthesis and release of neurotransmitters can either be catalyzed by the gut microbiota through food metabolism or by both of these methods [119]. A study reported that butyrate and propionate, produced by colonic enterochromaffin (EC) cells and serum, regulate the production of host 5-HT [139]. EC cells produce certain neuroactive metabolites, such as histamine, tryptophan, and PYY. Some gut microbes directly affect vagal nerve signaling, which stimulates the dorsal motor nucleus of the vagus (DMV) [140]. AD has also been linked to stress. Any type of stress can lead to psychological anguish, which may be accompanied by oxidative damage and inflammation, either from the outside or from within. Psychological stress leads to activation of the HPA axis, causing the release of glucocorticoids into the bloodstream, which then enters the brain through the BBB and activates the glucocorticoid receptor in humans and mineralocorticoid receptor in mice [141,142].

Hyperactivation of the HPA axis, induced by inflammatory processes and dysbiosis, can be prevented by probiotics [143]. It has been reported that administration of *Lactobacillus rhamnosus* reduces anxiety-like behavior and corticosterone levels in non-stressed mice [144]. A study using a mouse model of chronic stress reported that *Bifidobacterium pseudocatenulatum* was administered to experimental subjects to improve their glucocorticoid sensitivity and reduce inflammation [145].

## 6. Studies Conducted Using Animal Models

The studies on strategies for amelioration of AD symptoms are summarized in Table 1. In a study, Wistar rats were divided into four groups: group I received saline and group II received galactose (120 mg/kg body weight). D-galactose was injected into group III animals for six weeks, followed by a concurrent 60-day dose of D-galactose and *L. plantarum* MTCC 1325 (12 × 10^8^ CFU/mL; 10 mL/kg body weight). After 60 days, the rats in group IV received *L. plantarum*. Later, it was discovered that the morphometric and behavioral changes were accompanied by a significant decrease in Ach levels in the AD group, along with the development of tangles and amyloid plaques. Although the cognitive problems in the AD group were addressed, Ach levels and histopathological features were restored to those of the control group after 60 days of therapy with *L. plantarum* [146].

According to Bonfili et al., an SLAB51 formulation (a combination of lactic acid bacteria and Bifidobacteria), which has been reported to affect various neuronal pathways and significantly slow the progression of AD in 3XTg-AD mice, exerts modulatory effects on the gut microbiota [147]. In a similar study, SLAB51 was administered in combination with water to a probiotic-treated group, whereas water alone was administered to the control group of 3Xtg-AD mice. *Streptococcus thermophilus*, *Bifidobacterium longum*, *Bifidobacterium breve*, *Bifidobacterium infantis*, *Lactobacillus acidophilus*, *L. plantarum*, *L. paracasei*, *L. delburueckii* susp. bulgaricus, and *L. brevis* were the nine strains constituting the SLAB51 formulation. The daily dose was 200 billion bacteria/kg. Superoxide dismutase and glutathione peroxidase are two antioxidant enzymes whose activities were markedly elevated after receiving SLAB51. In mice with untreated AD, levels of modified base 8-oxodg peaked at 12 weeks of age, but treatment with SLAB51 returned the levels to baseline [148]. The effects of probiotics on memory and oxidative stress indicators were studied using rats as an AD model. The oxidative stress biomarkers, such as elevated malondialdehyde levels and superoxide dismutase activity, improved in the Alzheimer’s probiotic group that received an intrahippocampal injection of -amyloid (Aβ1–42) and probiotics supplementation (1 × 10^10^ CFU/g) for 8 weeks. *L. acidophilus*, *L. fermentum*, *B. lactis*, and *B. longum* were some of the available probiotics that were given out [149]. By lowering the amounts of soluble hippocampus Aβ1–42, presenilin 1 protein, and phosphorylated tau, *Bifidobacterium breve* MCC1274 supplementation in wild-type (WT) mice reduced the AD-related pathologies. In addition, it also reduced neuroinflammation and increased synaptic proteins [150]. Abraham et al. reported an increase in *Lactobacillus reuteri* levels in the gut and NRF-2 in the liver [151]. Teglas et al. subjected mice with AD to probiotic supplementation and found that intermittent treadmill running [152] had beneficial effects on elevating the antioxidant status in AD mice. Furthermore, they also found that both exercise and probiotics had no effect on mitochondrial density and protein synthesis-associated pathways. By encouraging the formation of indole-3-aldehyde and indole-3-propionic acid, *Lactobacillus reuteri* can reduce neuroinflammation in astrocytes. Subsequently, they pass through the BBB [153]. Due to its ability to promote the expression of cytoprotective, anti-inflammatory, and antioxidant genes, NRF-2 is crucial for neuronal defense [154,155].

Saffron contains curcumin, which has neuroprotective properties. Patel et al., reported that curcumin in combination with *Lactobacillus rhamnosus* may act as an adjuvant to improve memory and learning and ameliorate antioxidant enzymes in mice with scopolamine-induced dementia [156].

The protective effects of *Bacillus subtilis* were further investigated by Cogliati et al., using a *Caenorhabditis elegans* AD model. *Bacillus subtilis*-colonized *C. elegans* strains CL2120 showed resistance to the behavioral impairments brought on by the production of pan-neuronal toxic peptide Aβ1–42. For *B. subtilis* to exert its anti-AD benefits, it must first develop a biofilm in the gut [157].

In a study involving probiotic (VSL#3) administration for two months, the microbiota of both WT and App^NL-G-F^ mice changed. Both mouse genotypes showed increased levels of serum SCFAs, lactate, acetate, and butyrate. In App^NL-G-F^ mice, increased lactate levels led to increased c-fos levels in the brain. C-Fos plays a critical role in lowering behavioral anxiety by modifying the stress response [158].

According to Kobayashi et al., the probiotic strains *Bifidobacterium breve* strain A1 and *Bifidobacterium infantis* reduced the deposition of Aβ, α-TNF, and IL-1 and increased the level of SOD in the hippocampus region of the brain in A-induced AD mice [159]. Administration of *Bifidobacterium* enhances cognitive performance and suppresses the expression of immune-reactive genes by raising plasma acetate levels in the hippocampus. It can be inferred that *Bifidobacterium* has the capacity to exert protective effects against neuroinflammation and regulate immunological response that develops as a result of Aβ toxicity in the brain tissue. Additionally, according to Desbonnet et al. *Bifidobacterium* has the capacity to reduce the toxicity caused by Aβ and normalize gene expression profiles, particularly BDNF, which enhances neuronal survival in AD [160]. In a recent study, *Clostridium butyricum* was intragastrically administered to APPswe/PS1dE9 (APP/PS1) transgenic mice for a period of four weeks. Proinflammatory cytokine production, microglial activation, Aβ load, gut microbiota composition, and butyrate concentrations were all examined. Treatment with *Clostridium butyricum* prevented cognitive impairment, Aβ deposition, microglia activation, and TNF-α and IL-1β production in the APP/PS1 mouse brain. After *Clostridium butyricum* treatment, the aberrant gut microbiota and butyrate levels were reversed. Butyrate therapy also reduced NF-kB p65 phosphorylation in BV2 microglia caused by Aβ, which in turn resulted in a decrease in CD11b and COX-2 levels [161]. In a related study, probiotic supplements were administered to a group of APP/PS1 transgenic mice (APP/PS1TG) along with exercise training, and it was found that these mice performed better in the Morris Maze Test than the control group, but also had lower levels of β-amyloid plaques in their hippocampi [151].

Mohammadi et al. in their study divided rats into four experimental groups and administered intraperitoneal injections of saline, LPS (1 mg/kg, single dose), or probiotics (10^9^ CFU/mL/rat) for 20 h, after receiving intravenous administration of maltodextrin (placebo) or probiotics (10^9^ CFU/mL/rat) orally for 14 consecutive days. Four hours after the injections, memory recall and neuroinflammatory indicators were evaluated. Following pretreatment with probiotics, the high levels of pro-inflammatory cytokines in the hippocampus that had been caused by systemic exposure to LPS were significantly reduced. The combination of *B. longum* R0175 and *L. helveticus* R0052 inhibited the detrimental effects of LPS on memory through the expression of BDNF [161].

Aβ-amyloid was injected intracerebroventricularly to cause AD in an animal model. Two groups of control rats received probiotics and water as the vehicle (pro + con). The other two groups of animals with AD were either administered probiotics (probiotics + AD) or the vehicle. Memory and spatial learning were examined using Morris Water Maze tests. Long-term potentiation (LTP) and fundamental synaptic transmission were assessed by analyzing the postsynaptic potentials (fEPSPs) in the hippocampus. LTP was suppressed in rats with AD, but fundamental synaptic transmission remained unaffected. However, probiotic therapy improved LTP in the probiotics + control group and restored it in the probiotics + AD group. In the probiotics + AD group, the probiotic treatment also corrected the balance between oxidative and antioxidative indicators. These results provide additional evidence of the beneficial effects of probiotics on synaptic plasticity in animal models of AD [142].

The results obtained by Mehrabadi et al. were in concordance with the previous work of Kobayashi et al. [162]. Rats were divided into five groups (n = 10 each group) to assess the protective effects rendered by probiotics: control, sham, AD group with Aβ1–40 intrahippocampal injection and no dietary plan, AD + probiotics group with Aβ1–40 intrahippocampal injection and receiving 2 g (10^10^ CFU) probiotics (*Lactobacillus reuteri*, *Lactobacillus rhamnosus*, and *Bifidobacterium infantis*) orally once a day for 10 weeks, and AD + rivastigmine group, with rivastigmine (0.6 mg/kg) being administered orally once a day for two weeks. The Morris Water Maze test analysis showed that the probiotic treatment considerably improved spatial memory. The probiotics also reduced the Aβ plaques in AD-stricken animals. Additionally, there was a drop in malondialdehyde and a rise in the SOD enzyme activity. Probiotics also decreased the level of inflammatory markers IL-1 and TNF-α in rat AD models [162]. In another study, mice treated with scopolamine to induce memory deficits were examined for protective benefits of *Lactobacillus pentosus* var. *plantarum* C29 isolated from kimchi. Morris Water and Y-Maze tests revealed that the strain C29 provided protective benefits against scopolamine-induced memory impairment. Furthermore, scopolamine injection decreased the expression of p-CREB and BDNF in the hippocampus, which was increased by the C29 strain [163]. In a study by Azm et al. 60 rats were divided into five groups. The control and control + probiotics groups received probiotics for a duration of 8 weeks, intrahippocampal injection was administered to the sham group, the Alzheimer’s group received intrahippocampal injection of β-amyloid (Aβ1–42), and the Alzheimer’s group treated with probiotics received 2 g (1 × 10^10^ CFU/g) of probiotics (*Bifidobacterium longum*, *L. fermentum*, *L. acidophilus* and *Bifidobacterium lactis*) for 8 weeks. The Morris Water Maze test showed that the probiotic-treated Alzheimer’s group had significantly better spatial memory, which included a lower escape latency. Similar to other studies, probiotic treatment improved the biomarkers of oxidative stress, such as malondialdehyde levels and SOD activity. Thus, by modifying the gut microbiota, probiotics can enhance memory deficiency and suppress the pathogenic mechanisms linked to AD [149].

**Table 1:** Summary of studies on strategies for amelioration of AD symptoms.

S.No.	AD Model	Findings	Reference
1.	Wistar rats	Administration of D-galactose induced degeneration of nerve cells. However, treatment with *L. plantarum* MTCC1325 restored the levels of Ach close to normal. Moreover, the histopathological features were found to be similar to control group when treated with the probiotic.	[146]
2.	3XTg-AD	Neurodegenerative process in AD takes place via oxidative stress and generation of reactive oxygen species. Treatment with SLAB51 restored the defensive mechanism against oxidative stress to basal levels.	[148]
3.	AD rat model (Intrahippocampal injection of Aβ)	The following probiotics in combination: *Lactobacillus acidophilus* 1688FL431–16LA02, *Lactobacillus fermentum* ME3, *Bifidobacterium lactis* 1195SL609–16BS01, and *Bifidobacterium longum* 1152SL593–16BL03 controlled oxidative stress which was evident from the reduced levels of MDA and increased activity of superoxide dismutase in the hippocampus. The probiotics supplementation improved spatial memory and learning.	[149]
4.	WT mice	Oral administration of *Bifidobacterium breve* MCC1274 reduced the levels of phosphorylation and decreased the soluble Aβ42 levels.	[150]
5.	AD-injected mice	Administration of *Lactobacillus reuteri* suppressed neuroinflammation in astrocytes.	[153]
6.	Scopolamine-injected mice	*Lactobacillus rhamnosus* administration increased the levels of antioxidant enzymes SOD, GPx, and CAT in tissues.	[156]
7.	*C. elegans*	*Bacillus subtilis* colonization in *C. elegans* CL2120 strains had anti-AD effects.	[157]
8.	App^NL-G-F^ mice	Supplementation of VSL#3 increased lactate production, which in turn increased c-fos levels that modulated stress response.	[158]
9.	Aβ _1–42_-induced mice	*B. longum*, *L. acidophilus,* and *B. bifidum* improved long term potentiation, memory, and spatial learning.	[142]
10.	Aβ-induced mice	*B. breve* strain A1and *B. infantis* decreased Aβ deposition, IL-1β and α-TNF increased the SOD level in brain.	[160]
11.	APPswe/PS1dE9 transgenic AD model (APP/PS1) mice and wild-type C57BL/6 (WT) mice	*Clostridium butyricum* WZMC1016 reduced the level of IL-1β and α-TNF in brain, increased the level of butyrate in feces, suppressed microglia activation, reduced COX-2 expression in brain, and decreased p-p65 levels in brain.	[161]
12.	Male APP/PS1 transgenic mice	*Bifidobacterium longum*, lysates of *Lactobacillus acidophilus* were given in combination with omega 3 fatty acids, vitamin B complex, and treadmill running at intervals. This led to reduction in αβ plaques in the hippocampus, increase in the microglial cells and levels of DNA repair enzyme OGG1 in the brain, and increase in cognition and CFU of *Lactobacillus reuteri* in the gut.	[151]
13.	Wistar rats	*Lactobacillus helveticus* R0052e, *Bifidobacterium longum* R0175 attenuated cognitive defects, increased the expression of BDNF, and decreased the circulating and hippocampal levels of proinflammatory cytokines.	[164]
14.	Wistar rats	*Lactobacillus reuteri*, *Lactobacillus rhamnosus, and Bifidobacterium infantis* reduced the levels of Aβ plaques, oxidative stress, improved the activity of superoxide dismutase, and also reduced the levels of neuroinflammatory markers.	[162]
15.	Male ICR mice	*Lactobacillus pentosus and Lactobacillus plantarum C29 increased memory and spatial learning along with the level of BDNF and cAMP response element binding protein in the hippocampus.*	[163]

## 7. Health Benefits of Probiotics Based on Human Studies

Probiotics have been shown to be effective in lowering the degenerative changes linked to a variety of neurodegenerative illnesses, including AD [165]. The studies on use of probiotics for amelioration of AD symptoms have been summarized in Table 2. Probiotic formulations containing *Lactobacillus fermentum*, *Bifidobacterium bifidum*, *Lactobacillus acidophilus*, and *Lactobacillus casei* were tested in patients with AD to determine their preventive effects. In this trial, 60 patients with AD were divided into two groups (n = 30 in each group), with the probiotic group receiving the formulation and the control group receiving milk. The trial was controlled, randomized, and double blinded. The study was conducted over the course of 12 weeks. An assessment of metabolic status revealed little to no impact on fasting plasma glucose, lipid profiles, oxidative stress biomarkers, and inflammation. Mental state assessments revealed that probiotics had a favorable impact on cognitive functioning [166].

A similar study was conducted by Agahi et al., who divided their participants into control and probiotic-treated groups. The probiotic blends that were evaluated included *Bifidobacterium bifidum*, *Lactobacillus acidophilus*, *Lactobacillus plantarum*, and *Lactobacillus fermentum* [167]. The probiotic-treated group received one probiotic pill daily. Patients’ cognition was evaluated using a memory test. Additionally, the serum level of inflammatory markers including cytokines (TNF-α, IL-6, and IL-10) and oxidative stress biomarkers like malondialdehyde, glutathione, and 8-hydroxy-2′-deoxyguanosine (MDA) was also assessed. All measurements were recorded before and after supplementation. Probiotic supplementation did not demonstrate protective effects in patients with severe AD.

The preventive effects of probiotics and selenium co-supplementation were observed in a study of 79 patients with AD. For 12 weeks, the patients received either a placebo or co-supplementation (selenium 200 g/day with probiotics containing 2 × 10^9^ CFU/day of *Bifidobacterium bifidum*, *Bifidobacterium longum*, and *Lactobacillus acidophilus*). The probiotic plus selenium-supplemented group significantly outperformed the placebo group in terms of brief mental state examination. The total glutathione and antioxidant capabilities also increased significantly. High-sensitivity C-reactive protein, serum triglyceride, very-low-density lipoprotein, low-density lipoprotein, and total cholesterol levels were all significantly lowered in the probiotic-supplemented group than in the selenium and placebo groups [168]. Leblhuber et al. examined the advantages of probiotic supplementation in 20 outpatients with AD. Gut microbiota inflammatory markers and immune activation biomarkers were analyzed before and after supplementation [169]. Activation of macrophages, which could aid in the dissolution of amyloid aggregates, was thought to be the source of an increase in kynurenine levels in blood following probiotic supplementation. The kynurenine to tryptophan ratio and neopterin concentrations were found to be correlated, indicating that dendritic cells and macrophages were activated.

Limitations of the reviewed studies are mentioned to some extent in the conclusion section, like that the psychological questionnaires used during clinical trials may have resulted in subjective biases and care must be taken while evaluating them. In addition, probiotic supplementation should be avoided in patients receiving immunosuppressive drugs, such as chemotherapy. The studies discussed were limited to a fixed duration of approximately 30–90 days. To clarify the therapeutic effects of probiotics over a longer period, clinical trials need to be planned and conducted over a prolonged duration.

## 8. Conclusions and Future Perspectives

As mentioned above, probiotics may offer protection against the neurodegenerative alterations linked to AD, as evidenced by research in animal models and clinical trials. Most of the studies included in this review concentrated on the advantages of *Lactobacillus* and *Bifidobacterium*. Each study reported a noticeable improvement in cognition and memory. We also emphasized how the indicators for oxidative stress had changed. The effectiveness of multi-strain formulations was found to be promising. Each strain must be administered at a dose of at least 10^9^ or 10^10^ CFU to have a positive impact on the test subjects. It was shown that two weeks for animals and four weeks for humans produced favorable results. *Lactobacillus casei*, *Lactobacillus acidophilus*, *Lactobacillus plantarum*, *Bifidobacterium longum*, and *Bifidobacterium infantis* are the most commonly used multistrain formulations for AD. Probiotics, such as those described above, are well recognized as highly beneficial for human health since they strengthen the immune system and offer protection from diseases caused by harmful bacteria. Probiotics alter the composition of the gut microbiota, which in turn alters how the gut and brain communicate via the gut–brain axis. The nervous system is strengthened by probiotics, which also reduces the pathophysiological alterations associated with AD. The psychological questionnaires used in clinical trials may have subjective biases. Therefore, care must be taken when interpreting evaluations during clinical studies. Probiotics are often classified by the American Food and Drug Administration (FDA) as safe bacteria. Currently, there are no data supporting the safety of *Clostridium*, *Lactobacillus*, *Bifidobacterium*, or *Streptococcus* spp. in patients with AD. However, probiotic administration should be avoided in patients receiving immunosuppressive medications such as chemotherapy. The risk of infection in the host organism can occasionally be increased by antibiotic resistance genes of probiotic bacteria that spread to other dangerous bacteria [170]. Numerous examples discussed in this review demonstrate how probiotics can slow the progression of AD. We can state with certainty that the diet of patients with AD should include probiotic supplements as they have no known negative effects. Further extensive research is required to link the gut microbiome to development of AD. Designing probiotic microorganism-based treatment plans after thorough investigation of microbial diversity may improve the quality of life of patients with AD.

## Figures and Tables

**Table 2:** Summary of studies on use of probiotics for amelioration of AD symptoms.

S.No.	Probiotics	Duration	Effects	Reference
1.	*Bifidobacterium bifidum*, *Lactobacillus fermentum*, *Lactobacillus casei*, and *Lactobacillus acidophilus*	12 weeks	Reduction in serum MDA and triglyceride levels. Improvement in MMSE score. Reduction in high-sensitivity C-reactive protein (hs-CRP).	[166]
2.	*Lactobacillus fermentum*, *Lactobacillus plantarum*, *Lactobacillus acidophilus*, *Bifidobacterium lactis*, *Bifidobacterium bifidum*, and *Bifidobacterium longum*	12 weeks	Increase in TYM score and cognitive function. Increase in serum GSH. Decrease in serum 8-OHdG.	[167]
3.	*Bifidobacterium bifidum*, *Bifidobacterium longum*, *Lactobacillus acidophilus*, and selenium	12 weeks	Reduction in serum hs-CRP and triglyceride. Increase in antioxidant and GSH. Improvement in MMSE score.	[168]
4.	*Lactobacillus casei* W56, *Lactobacillus acidophilus* W22, *Lactococcus lactis* W19, *Bifidobacterium lactis* W52, *Lactobacillus plantarum* W62, *Lactobacillus paracasei* W20, *Bifidobacteium lactis* W51, *Bifidobacterium bifidum* W23	28 days	Reduction in fecal zonulin and Prausnitzii. Increase in faecalibacterium and serum concentration of kynurenine. Increase in concentration of neopterin and nitrite. Reduction in serum hs-CRP.	[169]
